# Magnetization control by angular momentum transfer from surface acoustic wave to ferromagnetic spin moments

**DOI:** 10.1038/s41467-021-22728-6

**Published:** 2021-05-10

**Authors:** R. Sasaki, Y. Nii, Y. Onose

**Affiliations:** 1 Department of Basic Science, University of Tokyo, Tokyo, 153-8902 Japan; 2 Institute for Materials Research, Tohoku University, Sendai, 980-8577 Japan; 3 PRESTO, Japan Science and Technology Agency (JST), Kawaguchi, 332-0012 Japan; 4 Present Address: RIKEN Center for Quantum Computing (RQC), Wako, 351-0198 Japan

**Keywords:** Magnetic properties and materials, Spintronics

## Abstract

Interconversion between electron spin and other forms of angular momentum is useful for spin-based information processing. Well-studied examples of this are the conversion of photon angular momentum and rotation into ferromagnetic moment. Recently, several theoretical studies have suggested that the circular vibration of atoms work as phonon angular momentum; however, conversion between phonon angular momentum and spin-moment has yet to be demonstrated. Here, we demonstrate that the phonon angular momentum of surface acoustic wave can control the magnetization of a ferromagnetic Ni film by means of the phononic-to-electronic conversion of angular momentum in a Ni/LiNbO_3_ hybrid device. The result clearly shows that the phonon angular momentum is useful for increasing the functionality of spintronic devices.

## Abstract (web summary)

Conversion of an external angular momentum, for example, from mechanical rotation or light into ferromagnetic moment has a long history. Here, Sasaki et al. demonstrate the conversion of phonon angular momentum, in ferromagnetic moment, potentially allowing for new types of control for spintronics.

## Introduction

**Fig. 1:**
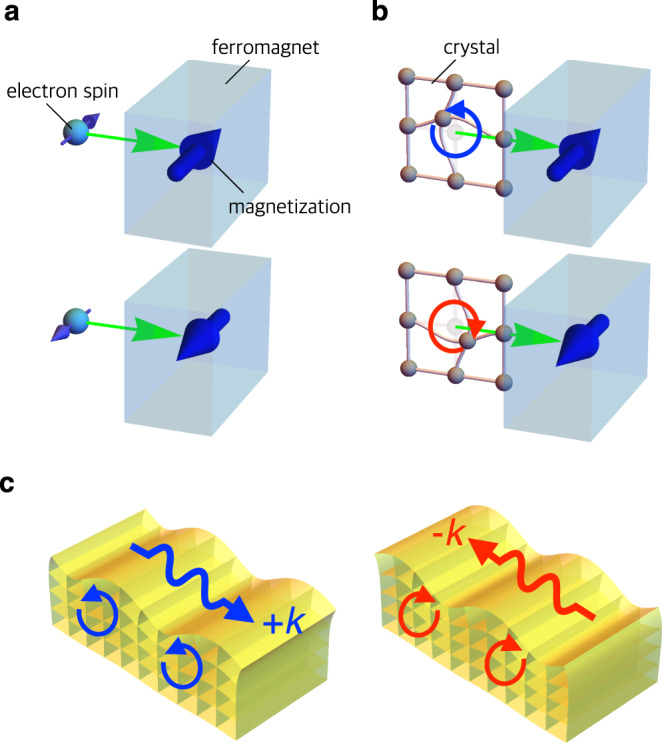
Concept of magnetization control by phonon angular momentum. **a** Schematic illustrations of magnetization control by electron spin injection. **b** Schematic illustrations of magnetization control by the injection of phonon angular momentum. **c** Schematic illustrations of phonon angular momenta in surface acoustic waves (SAWs). The direction of phonon angular momentum of SAW depends on the sign of wave vector *k*.

Angular momentum is conserved when a system has rotational symmetry. While this law is, strictly speaking, broken in crystals, approximate conservation remains valid in the microscale range^1^. For example, when a spin-polarized electrical current is injected into a microscale ferromagnet, the spin angular momentum of the conduction electrons is transferred to ferromagnetic localized moment (Fig. 1a). This mechanism is used to control magnetic storage in magnetoresistive random access memories^2^. The angular momentum of a rigid body rotation^3^ and photon^4,5^ can be also used to control the magnetization. One might wonder whether it is possible to control the magnetization via the angular momentum transfer from phonons^6–9^ (Fig. 1b).

The phonon angular momentum is activated by the breaking of time-reversal or spatial-inversion symmetry^6,10–14^. In time-reversal symmetry-broken ferromagnets, the polarization of a transverse acoustic wave (low-energy phonon mode) is observed to rotate while propagating along the magnetization direction^15^, which indicates an eigenstate with circular polarization. Similar circularly polarized phonons are also observed in spatial inversion symmetry-broken chiral materials, corresponding to the phonon version of natural activity^16^. The phonon angular momentum is also emergent on the surface of a substance. A Rayleigh-type surface acoustic wave (SAW) has elliptically polarized displacement^17^, which indicates that the phonon angular momentum is finite (Fig. 1c). The angular momentum is parallel to the vector product of the SAW wave vector *k* and surface normal vector, and it shows a sign change when *k* is reversed^18^. Here, we use the SAW current to demonstrate the conversion from phonon angular momentum to magnetization.

## Results

### Nonreciprocal propagation of surface acoustic wave

**Fig. 2:**
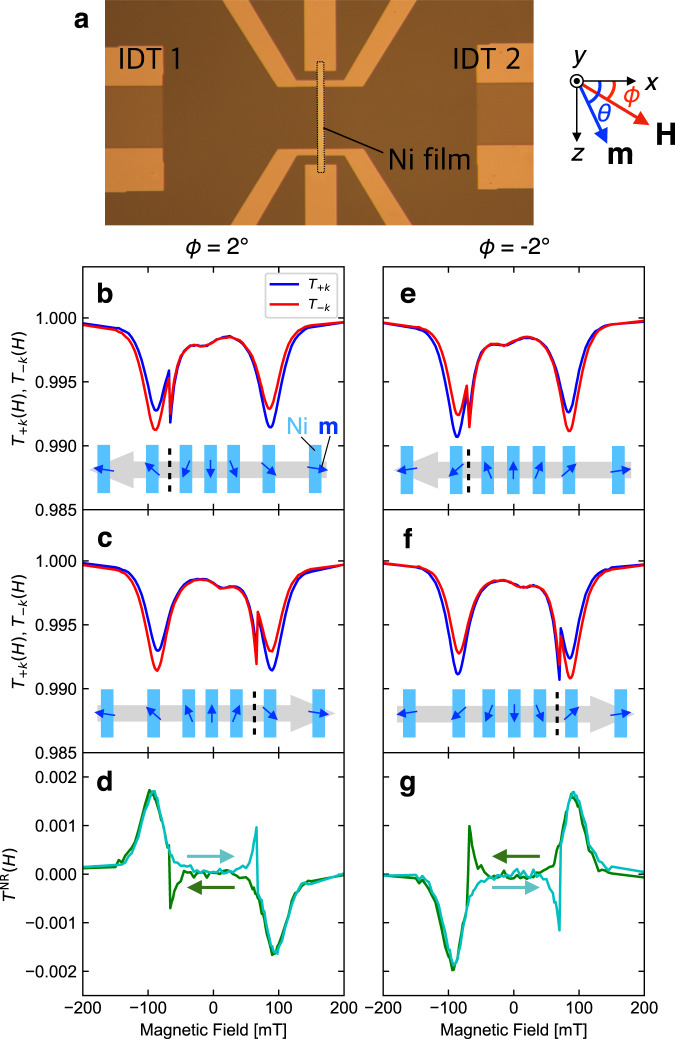
Nonreciprocal SAW propagation. **a** An optical micrograph of the SAW device used in this work. Two interdigital transducers (IDT 1 and IDT 2) and a Ni film with six electrodes were formed on a LiNbO_3_ substrate. The dotted rectangle emphasizes the Ni film. The right sketch illustrates the *xyz*-coordinate system, the angle *ϕ* between the *x*-axis and magnetic field vector **H**, and the angle *θ* between the *x*-axis and magnetization vector **m**, which are used for the explanation. **b**, **c** The magnetic field dependence of the SAW transmission along +*x* and −*x* directions (*T*_+*k*_(*H*), *T*_−*k*_(*H*)) in decreasing (**b**) and increasing (**c**) field at *ϕ* = 2^∘^. The insets illustrate the inferred magnetization directions in the Ni film. Gray arrow and black dashed line represent the sweep direction of the magnetic field and the magnetization *θ* flop field, respectively. **d** Nonreciprocity of the SAW transmission *T*^NR^(*H*) = *T*_+*k*_(*H*) − *T*_−*k*_(*H*) in decreasing and increasing field at *ϕ* = 2^∘^. **e**, **f** The magnetic field dependence of the SAW transmission in decreasing (**e**) and increasing (**f**) the field at *ϕ* = −2^∘^. **g** *T*^NR^(*H*) in decreasing and increasing field at *ϕ* = −2^∘^.

Figure 2a shows the SAW device used in this work. This device is composed of a piezoelectric LiNbO_3_ substrate, two interdigital transducers (IDTs) and a ferromagnetic Ni film^19–22^. The *xyz*-coordinate system is defined as shown in the right panel. To understand the coupling between the SAW and ferromagnetism, we demonstrate how the magnetization direction of the Ni film affects the SAW propagation. Figure 2b, c, e, f shows the SAW transmission in magnetic fields nearly parallel to the *x*-axis. The magnetic field angle *ϕ* is slightly tilted (*ϕ* = 2^∘^) to *z* direction from the *x*-axis in Fig. 2b, c whereas the tilted direction is reversed in Fig. 2e, f. The magnetic field increased from −400 to 400 mT (decreased from 400 to −400 mT) during the measurements shown in Fig. 2c, f (Fig. 2b, e). Before discussing the SAW transmission, let us explain the variation of magnetization in the magnetic fields. The insets illustrate the expected magnetization direction in the magnetic field sweep. Considering the shape anisotropy of the Ni film, the easy and hard axes are the *z*- and *y*-axis, respectively. In this case, the magnetization variation is very sensitive to the tilt direction and the sign of the magnetic field variation. In decreasing the magnetic field at *ϕ* = 2^∘^ (Fig. 2b), the tilt angle of the magnetization *θ* is positive, and the magnitude increases. At zero magnetic field, the magnetization points along the +*z* direction. When the magnetic field changes its sign, the magnetic state with negative *θ* is more energetically stable. Therefore, the sign of *θ* is abruptly reversed at some negative field, which is denoted as *θ* flop. In an increasing field (Fig. 2c), the magnetization points to the −*z* direction at zero magnetic field and *θ* flop shows up at a positive magnetic field. For the *ϕ* = −2^∘^ measurements (Fig. 2e, f), the magnetization shows similar variation but the sign of *θ* and the magnetization direction at zero field are opposite.

Next, we discuss the SAW transmission. We measured the transmission intensity from IDT1 to IDT2 *T*_+*k*_(*H*) and that from IDT2 to IDT1 *T*_−*k*_(*H*) at various magnetic fields *H* (see Supplementary Information for precise definitions of *T*_+*k*_(*H*) and *T*_−*k*_(*H*)). For all the measurements, *T*_+*k*_(*H*) and *T*_−*k*_(*H*) showed a broad dip around ± 90 mT. This was ascribed to the ferromagnetic resonance (FMR) excitation by the acoustic wave via magnetoelastic coupling^19,20^. The discontinuous changes at −70 mT in Fig. 2b, e and those at +70 mT in Fig. 2c, f were caused by the *θ* flops mentioned above. Importantly, the intensity of acoustically excited FMR depends on the propagation direction of the SAW. We plot the difference of transmittance *T*^NR^(*H*) = *T*_+*k*_(*H*) − *T*_−*k*_(*H*) at *ϕ* = +2^∘^ and −2^∘^ in Fig. 2d and g, respectively. *T*^NR^(*H*) was independent of the magnetic field sweep direction except for the region around the *θ* flop fields, but it showed the opposite sign when either the sign of the field or *ϕ* was reversed. This phenomenon is denoted as nonreciprocal SAW propagation induced by the simultaneous breaking of time-reversal and spatial inversion symmetries^21,22^. In this case, the ferromagnetism and surface break the time reversal and spatial inversion symmetries, respectively. The nonreciprocity originates microscopically from the different polarizations of the +*k* and −*k* modes. As mentioned above, the SAW has elliptical polarization, and the rotational direction is reversed by the reversal of *k*. On the other hand, FMR can be excited only by an effective field with right-handed circular polarization. These effects were the origin of the difference in the acoustic FMR intensity between +*k* and −*k* SAWs. Conversely, the ratio of nonreciprocity to absorption reflects the ellipticity of the SAW polarization.

### Numerical demonstration of magnetization control

**Fig. 3:**
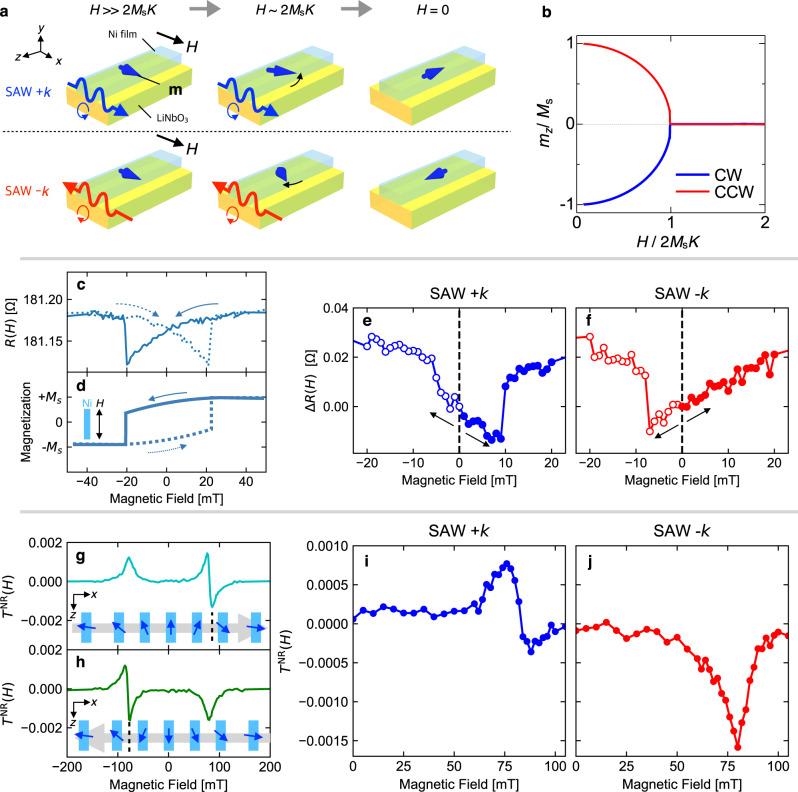
Demonstration of magnetization control by phonon angular momentum. **a** Schematic illustrations of the magnetization **m** control by phonon angular momentum of SAW. Here, 2*M*_s_*K* is anisotropy field. The application of SAW current with the wave vector parallel (+*k*) or anti-parallel (−*k*) to the *x*-axis (CW polarization or CCW polarization) during the decrease of magnetic field controls the magnetization direction at *H* = 0. **b** The numerically calculated magnetization evolution under the application of SAW currents along + *x* and − *x*. **c** The magnetoresistance of Ni thin film as a function of magnetic field along the *z*-axis (*ϕ* = 90^∘^). **d** The illustration of magnetization curves inferred by the resistance measurement shown in **c**. **e**, **f** The magnetic field dependence of the resistance difference Δ*R*(*H*) = *R*(*H*) − *R*(0) at *ϕ* = 90^∘^ after the poling procedure with SAW currents along +*x* (**e**) and −*x* (**f**) directions. The dashed lines and the arrows indicate the initial field (0 mT) and field sweep directions, respectively. **g**, **h** The magnetic field dependence of the SAW transmission nonreciprocity *T*^NR^(*H*) = *T*_+*k*_(*H*) − *T*_−*k*_(*H*) in decreasing (**g**) and increasing (**h**) field. The magnetic field direction is very close to the *x*-axis. The angle *ϕ* is positive and the magnitude is less than 0.5^∘^ (see text). The insets illustrate the magnetization direction in the film inferred by the nonreciprocity. Gray arrow and black dashed line represent the sweep direction of the magnetic field and the magnetization *θ* flop field, respectively. **i**, **j** The magnetic field dependence of *T*^NR^(*H*) measured with increasing magnetic field from 0 mT after the magnetic poling with SAW current along +*x* (**i**) and −*x* (**j**) directions.

Now we discuss the inverse effect of the nonreciprocal propagation. Intuitively, the inverse effect would be control of the time reversal symmetry or magnetization by using the spatial inversion symmetry-broken surface state and the unidirectional SAW flux. To demonstrate this, we consider the magnetization variation in the field decreasing process with SAW flux after applying a strong magnetic field along the *x*-axis (Fig. 3a). In this case, the magnetization points along the *x*-axis at first, and then it is tilted in either the +*z* or −*z* direction due to the shape anisotropy. The SAW flux along the +*x* or −*x* direction is expected to control whether the magnetization is tilted to the +*z* or −*z* direction.

To confirm this conjecture we have performed a numerical simulation. The magnetization should vary following the Landau Lifshitz Gilbert (LLG) equation expressed as

1 $$\frac{{\rm{d}}{\bf{m}}}{{\rm{d}}t}=-\gamma {\bf{m}}\times {{\bf{H}}}_{{\rm{eff}}}+\frac{\alpha }{{M}_{{\rm{s}}}}{\bf{m}}\times \frac{{\rm{d}}{\bf{m}}}{{\rm{d}}t},$$

where **m** = (*m*_*x*_, *m*_*y*_, *m*_*z*_), *γ*, *α*, and *M*_s_ are a uniform magnetization vector, the gyromagnetic ratio, Gilbert damping, and saturation magnetization, respectively. ${{\bf{H}}}_{{\rm{eff}}}=\left(-\frac{\partial F}{\partial {m}_{x}},-\frac{\partial F}{\partial {m}_{y}},-\frac{\partial F}{\partial {m}_{z}}\right)$ is the effective magnetic field, in which free energy *F* is composed of Zeeman energy *F*_Z_ = −**m** ⋅ **H** (**H** is the external magnetic field vector), magnetic anisotropy *F*_a_, and a magentoelastic field *F*_me_. For simplicity, we assume uniaxial magnetic anisotropy ${F}_{{\rm{a}}}=-K{m}_{z}^{2}$ (*K* is constant) and the magnetoelastic coupling energy for a polycrystal given by^20,23^

2 $${F}_{{\rm{me}}}=b\mathop{\sum}\limits_{i}{m}_{i}^{2}{e}_{ii}+b\mathop{\sum}\limits_{i\ne j}{m}_{i}{m}_{j}{e}_{ij},$$

where *b* is the magneto-elastic coupling constant, and *m*_*i*_ and *e*_*ij*_ are the *i*th component of the magnetization and the strain tensor, respectively. For a Rayleigh-type SAW propagating along the *x* direction, non-vanishing components of the strain tensor are *e*_*xx*_, *e*_*xy*_, and *e*_*yy*_. By introducing spin moment **S** = (*S*_*x*_, *S*_*y*_, *S*_*z*_) = −**m**/*gμ*_B_, *S*_±_ = *S*_*x*_ ± *iS*_*y*_, and *e*_*x*±_ = *e*_*xx*_ ± 2*ie*_*xy*_, *F*_me_ can be reduced to

3 $${F}_{{\rm{me}}}=\frac{b{(g{\mu }_{{\rm{B}}})}^{2}}{2}\left[{S}_{x}({S}_{+}{e}_{x-}+{S}_{-}{e}_{x+})+2{S}_{y}^{2}{e}_{yy}\right].$$

Here, *g* and *μ*_B_ are the *g*-factor and Bohr magneton, respectively. This formula clearly shows the angular momentum transfer from the phononic to magnetic systems (see Supplementary Information for details). Figure 3b shows the calculated magnetic field variation of *m*_*z*_ under a SAW current. We assumed the *x* and *y* components of SAW-induced dynamical displacement to be purely circular as ${u}_{x}(t)={u}_{0}\cos (kx-\omega t)$ and ${u}_{y}(t)=-{\rm{sgn}}(k){u}_{0}\sin (kx-\omega t)$, and neglected decay along the *y* direction for simplicity. The sign of the rotational motion depends on the sign of the wave vector *k*. Then the relevant strains are expressed as ${e}_{xx}(t)=\frac{\partial {u}_{x}}{\partial x}=-k{u}_{0}\sin (kx-\omega t)$ and ${e}_{xy}(t)=\frac{1}{2}\left(\frac{\partial {u}_{x}}{\partial y}+\frac{\partial {u}_{y}}{\partial x}\right)=-(| k| {u}_{0}/2)\cos (kx-\omega t)$. The other parameters used for the numerical calculation are shown in Methods. At *t* = 0, a strong magnetic field is applied along the *x*-axis, and we assumed **m** = (*M*_s_, 0, 0). Then the magnetic field is slowly decreased as ${H}_{0}\exp (-t/{t}_{0})$. *m*_*z*_ evolves below the anisotropy field 2*M*_s_*K*. Importantly, the *m*_*z*_ direction depends on whether the SAW polarization is clockwise (CW) or counter-clockwise (CCW), which correspond to SAWs propagating along the +*x* and −*x* directions, respectively. This demonstrates deterministic control of magnetization by selecting SAW direction. The mechanism involved seems to be the damping torque which is dependent on the direction of polarization rotation. Circular polarization induces rotational motion of magnetization in the *xy* plane. The damping torque due to the rotational motion *τ*_*z*_ = (**m**×d**m**/d*t*)_*z*_ is parallel or antiparallel to the *z*-axis, and the sign depends on the direction of rotation. Note that these results are independent of the phase of the SAW (see Supplementary Information) and the effect is thus different from precessional switching^24^.

### Experimental demonstration of magnetization control

Next, we describe the corresponding experimental demonstration of magnetization control by using the phonon angular momentum. We first applied the magnetic field as large as 400 mT along the *x*-axis and set the SAW current along either the +*x* or −*x* direction (from IDT1 to IDT2 or IDT2 to IDT1), of which the excitation power and frequency were 25 dBm and 2.906 GHz, respectively. To be precise, the magnetic field seemed to be slightly tilted in the +*z* direction but the angle between the magnetic field and the *x*-axis was less than 0. 5^∘^ (see Supplementary Information). Then we decreased the magnetic field to zero at a rate of 0.01 T s^−1^. Hereafter, we call the sequence of these operations “poling procedure”. To detect the magnetization direction after the poling procedure, we used the magnetoresistance. Figure 3c shows the magnetoresistance *R*(*H*) measured in magnetic fields parallel to the electric current in the Ni film. It shows a butterfly-shaped hysteresis loop. Since the magnetoresistance is dependent on the magnitude of *m*_*z*_, the magnetization curve with a finite coercive force can be inferred as shown in Fig. 3d. The magnetization should be saturated in the high field region where *R*(*H*) is constant. The decreases of magnetoresistance before the discontinuous jump in Fig. 3c corresponds to the gradual decrease of ∣*m*_*z*_∣ before the flip. One can distinguish the magnetization state at zero magnetic field (**m**∣∣ +*z* or **m**∣∣ −*z*) by measuring the magnetoresistance along the *z*-axis. When the resistance decreases with increasing magnetic field from *H* = 0 and discontinuously increases at a certain magnetic field, the magnetization should have pointed in the −*z* direction at zero field. On the other hand, when it increases continuously without any discontinuity, the magnetization direction was opposite. If the magnetic field is decreased from *H* = 0, the field dependences of resistance for the magnetic states of **m**∣∣ +*z* and **m**∣∣ −*z* should be reversed. To probe the magnetization in this way, we rotated the device by 90^∘^ around the *y*-axis after the poling procedure and measured the field dependence of the resistance along the *z*-axis while increasing or decreasing the field from 0 mT. Note that the positive magnetic field points the +*z* direction after the rotation.

Figure 3e and f shows the magnetic field dependence of Δ*R*(*H*) = *R*(*H*) − *R*(0) after the field poling with SAW currents along the +*x* and −*x* directions, respectively. In the case of the SAW current along the +*x* direction, the resistance decreased at first with increasing the field from 0 mT and showed a discontinuous increase around 10 mT whereas it increased with decreasing magnetic field from 0 mT almost continuously. Conversely, after the poling with the SAW current along the −*x* direction, the resistance increased (decreased) with increasing (decreasing) magnetic field from 0 mT. A discontinuous increase of resistance was observed only when the magnetic field was decreased. These results demonstrate that the SAW currents along the +*x* and −*x* direction aligned the magnetization along the −*z* and +*z* directions, respectively. Thus, control of the magnetization by means of the SAW current was realized. The SAW direction dependence of the controlled magnetization cannot be explained by the effects of some static strains or other trivial effects, but it is naturally explained by the effects of phonon angular momentum transfer. The magnetization direction was opposite to the phonon angular momentum direction, which is consistent with the numerical simulation shown in Fig. 3b. While the slight tilting magnetic field induces the energy imbalance between **m**∣∣ +*z* and **m**∣∣ −*z* states, the angular momentum transfer from SAW could reversely control the magnetization overcoming the energy imbalance. For more details about the input power and angle *ϕ* dependence, see the Supplementary Information.

To confirm the experimental demonstration of magnetization control, we also probed the magnetization after the poling procedure by using nonreciprocal SAW transmission *T*^NR^(*H*). Figure 3g and h shows *T*^NR^(*H*) in increasing and decreasing the magnetic field almost parallel to the *x*-axis, respectively. Precisely speaking, the angle of the magnetic field *ϕ* seems to have slightly deviated from zero to the positive side (*ϕ* < 0. 5^∘^) because the magnetic field dependence of *T*^NR^(*H*) was similar to the case of *ϕ* = 2^∘^. The magnetic hysteresis became larger than that at *ϕ* = 2^∘^, and the discontinuous sign change overlapped with the dip or peak. One can probe the magnetization at zero magnetic field by using the magnetic field dependence of *T*^NR^(*H*). If *T*^NR^(*H*) shows a simple dip as the magnetic field is increased from zero, the magnetization direction at 0 mT was parallel to +*z*. On the other hand, if *T*^NR^(*H*) shows a peak followed by a discontinuous sign change, the magnetization pointed in the −*z* direction at zero field. Figures 3i and j show *T*^NR^(*H*) after the poling with SAW currents along the +*x* and −*x* directions, respectively. As shown in Fig. 3i, *T*^NR^(*H*) showed a peak and a sign change around 80 mT after poling with the SAW current along the +*x* direction. Therefore, the magnetization pointed in the −*z* direction at *H* = 0. On the other hand, *T*^NR^(*H*) after poling with a SAW current along the −*x* direction showed a simple dip, indicating that the magnetization pointed along the +*x* direction at *H* = 0. These results are consistent with the magnetoresistance measurements. The same measurement was also performed at a small negative angle, and the same result was found (see Supplementary Information).

## Discussion

We have demonstrated magnetization control by the angular momentum transfer from a SAW to ferromagnetic spin moments. However, it should be noted that the volume fraction of the controlled magnetization seems to be less than 100 % in the experiments. The magnitude of the resistance discontinuities in Fig. 3e and f is nearly 40 % of that in Fig. 3c. The magnetic field variations shown in Fig. 3i and j is weaker than those in Fig. 3g and h. The volume fraction of the controlled ferromagnetic domain seems to be several tens of percent. A number of related experimental and theoretical studies have already been reported. While the interconversion between the mechanical rotation and spin moment is known as the Einstein-de Haas effect and the Barnet effect^3,25^, the present result demonstrates the conversion from the angular momentum of a microscopic phonon excitation. More recently, Kobayashi et al. reported the generation of alternating spin current from a SAW^26^. This phenomenon is qualitatively different from the present result, which originates from the time-independent angular momentum in a SAW. The concept of phonon angular momentum has recently been theoretically developed in the spintronics field^6,6–14^. The present results experimentally demonstrate an important functionality of angular momentum, the conversion to ferromagnetic spin moments, which shows the validity of phonon angular momentum. If the effective magnetic field produced by SAW becomes large enough, even SAW application alone without the help of magnetic field can control the magnetization. This might be achieved by optimization of the devise structure and material. Recent literature reported that similar optimization makes the closely related phenomenon of nonreciprocal SAW propagation gigantic^22,27^. The magnetization manipulation by SAW seems useful to transfer the information carried by the SAW or microwave signal to the magnetic storage. In this sense, it is expected to provide a bridge between telecommunication technology and spintronics because SAW devices are indispensable in contemporary telecommunications technology.

## Methods

### Device fabrication

The SAW device in this work was fabricated by electron beam lithography. The device substrate was Y-cut LiNbO_3_ and the SAW propagation direction was along the Z-axis of the substrate. Both the IDTs and electrodes were made of Ti (5 nm) and Au (20 nm). One IDT had 200 pairs of 100 μm fingers, and the distance between two IDTs was 500 μm. The finger width and spacing of the IDTs were both 300 nm. The corresponding wavelength and frequency were 1.2 μm and 2.9 GHz, respectively. A Ni film was sputtered between two IDTs on the LiNbO_3_ substrate and was connected to six electrodes for resistance measurement. The thickness, width, and length of the Ni film were 30 nm, 10 μm, and 175 μm, respectively. After the Ni film was sputtered, the device was kept at 200 ^∘^C for 30 min to eliminate strain in the Ni film arising from the sputtering process^28^.

### Measurements of SAW transmission and resistance

All of the measurements in this work were done at 100 K. The SAW transmission was measured with a vector network analyzer. The microwave power was 10 dBm in Fig. 2b-g and 3g and h, and was -10 dBm in Fig. 3i and j. The magnetoresistance was measured using a lock-in amplifier with a frequency of 11.15 Hz.

### Numerical simulations

The LLG equation was numerically solved by using Mathematica. We set realistic values for the coefficients used in the calculation. The saturation magnetization and Gilbert damping coefficient were *M*_s_ = 370 kA m^−3^^23^ and *α* = 0.064^29^, respectively. The magnetic anisotropy constant was assumed to be $K{M}_{{\rm{s}}}^{2}=1.63\times 1{0}^{4}$ N m^−2^ for reproducing a flipping magnetic field of 2*KM*_s_ ≃ 0.088 T. The strain amplitude and frequency of the SAW were *e*_0_ = ∣*k*∣*u*_0_ = 2 × 10^−6^ and *f* = *ω*/2*π* = 2.9 GHz, respectively. The magnetoelastic coupling constants *b* were given by the average of *b*_1_ and *b*_2_, where ${b}_{1}{M}_{{\rm{s}}}^{2}=6.20\times 1{0}^{6}$ N m^−2^ and ${b}_{2}{M}_{{\rm{s}}}^{2}=4.30\times 1{0}^{6}$ N m^−2^^23^. The time constant for the decrease of magnetic field was *t*_0_ = 1 *μ*s.

## Supplementary information

Supplementary Information

Peer Review File

## Data Availability

The data that support the findings of this study are available from the corresponding author upon reasonable request.

## Code availability

The code that support the findings of this study are available from the corresponding author upon reasonable request.

## References

[1] Otani Y, Shiraishi M, Oiwa A, Saitoh E, Murakami S (2017). Spin conversion on the nanoscale. Nat. Phys..

[2] Brataas A, Kent AD, Ohno H (2012). Current-induced torques in magnetic materials. Nat. Mater..

[3] Barnett SJ (1915). Magnetization by rotation. Phys. Rev..

[4] Kimel AV (2005). Ultrafast non-thermal control of magnetization by instantaneous photomagnetic pulses. Nature.

[5] Stanciu CD (2007). All-optical magnetic recording with circularly polarized light. Phys. Rev. Lett..

[6] Zhang L, Niu Q (2014). Angular momentum of phonons and the Einstein-de Haas effect. Phys. Rev. Lett..

[7] Garanin DA, Chudnovsky EM (2015). Angular momentum in spin-phonon processes. Phys. Rev. B.

[8] Rückriegel A, Streib S, Bauer GEW, Duine RA (2020). Angular momentum conservation and phonon spin in magnetic insulators. Phys. Rev. B.

[9] Hamada M, Murakami S (2020). Conversion between electron spin and microscopic atomic rotation. Phys. Rev. Res..

[10] Zhang L, Niu Q (2015). Chiral phonons at high-symmetry points in monolayer hexagonal lattices. Phys. Rev. Lett..

[11] Hamada M, Minamitani E, Hirayama M, Murakami S (2018). Phonon angular momentum induced by the temperature gradient. Phys. Rev. Lett..

[12] Holanda J, Maior DS, Azevedo A, Rezende SM (2018). Detecting the phonon spin in magnon-phonon conversion experiments. Nat. Phys..

[13] Zhu H (2018). Observation of chiral phonons. Science.

[14] Chen X (2019). Entanglement of single-photons and chiral phonons in atomically thin WSe_2_. Nat. Phys..

[15] Matthews H, LeCraw RC (1962). Acoustic Wave Rotation by Magnon-Phonon Interaction. Phys. Rev. Lett..

[16] Pine AS (1970). Direct observation of acoustical activity in *α* quartz. Phys. Rev. B.

[17] Landau, L. et al. Theory of Elasticity: Volume 7. Course of theoretical physics (Elsevier Science, 1986).

[18] Long Y, Ren J, Chen H (2018). Intrinsic spin of elastic waves. Proc. Natl Acad. Sci. USA.

[19] Weiler M (2011). Elastically driven ferromagnetic resonance in nickel thin films. Phys. Rev. Lett..

[20] Dreher L (2012). Surface acoustic wave driven ferromagnetic resonance in nickel thin films: Theory and experiment. Phys. Rev. B.

[21] Sasaki R, Nii Y, Iguchi Y, Onose Y (2017). Nonreciprocal propagation of surface acoustic wave in Ni/LiNbO_3_. Phys. Rev. B.

[22] Tateno S, Nozaki Y (2020). Highly nonreciprocal spin waves excited by magnetoelastic coupling in a Ni/Si bilayer. Phys. Rev. Appl..

[23] Chikazumi, S. & Graham, C. D.*Physics of Ferromagnetism 2e*. 94 (Oxford University Press on Demand, 2009).

[24] Thevenard L (2016). Precessional magnetization switching by a surface acoustic wave. Phys. Rev. B.

[25] Einstein, A. & de Haas, W. J. Experimenteller nachweis der ampereschen molekularstrome. *Verh. d. Deutsch. Phy. Ges*. 152–170 (1915).

[26] Kobayashi D (2017). Spin current generation using a surface acoustic wave generated via spin-rotation coupling. Phys. Rev. Lett..

[27] Xu, M. et al. Nonreciprocal surface acoustic wave propagation via magneto-rotation coupling. *Science Advances *. **6** (2020). 10.1126/sciadv.abb1724 PMC7413730 32821833

[28] Wiegert R, Levy M (2001). Temperature dependence and annealing effects on magnetoelastic SAW attenuation and magnetoresistance in Ni films. IEEE Trans. Magn..

[29] Platow W, Anisimov AN, Dunifer GL, Farle M, Baberschke K (1998). Correlations between ferromagnetic-resonance linewidths and sample quality in the study of metallic ultrathin films. Phys. Rev. B.

