# Supplementary material for: Magnetization control by angular momentum transfer from surface acoustic wave to ferromagnetic spin moments
Source: Nat Commun. 2021 May 10;12:2599. doi: 10.1038/s41467-021-22728-6 (PMC8110840; doi:10.1038/s41467-021-22728-6)
Supplement: Supplementary file 1 — Supplementary Information [file 41467_2021_22728_MOESM1_ESM.pdf]

Supplementary Information for "Magnetization control by angular momentum transfer from surface acoustic wave to ferromagnetic spin moments"

R. Sasaki,[1,*] Y. Nii,[2,3] and Y. Onose[2]

[1]*Department of Basic Science, University of Tokyo, Meguro-ku, Tokyo 153-8902, Japan*

[2]*Institute for Materials Research, Tohoku University, Sendai 980-8577, Japan*

[3]*PRESTO, Japan Science and Technology Agency (JST), Kawaguchi 332-0012, Japan*

I. THEORY FOR ANGULAR MOMENTUM TRANSFER FROM PHONON TO SPIN MOMENT

In this section, we theoretically show how the magnetoelastic coupling transfers the angular momentum from the elastic to the magnetic system in the Ni thin film of the SAW device. Since the thickness of magnetic Ni film is much smaller than the wavelength of the SAW, we here neglect the depth dependence of the SAW amplitude and regard it as the circularly polarized plane wave in the Ni film. The SAW excites the ferromagnetic resonance by means of magnetoelastic coupling. We show that this process can be viewed as the angular momentum transfer from the elastic system to the magnetic system.

The free energy is

$$F_{\text{tot}} = F_{\text{el}} + F_{\text{mag}} + F_{\text{me}}, \tag{I.1}$$

where F_{el}, F_{mag}, and F_{me} are free energy for the elastic system, magnetic system, and magnetoelastic system, respectively. As shown in Eq. (2) in the main text, F_{me} can be given by

$$F_{\text{me}} = b \sum_i m_i^2 e_{ii} + b \sum_{i \neq j} m_i m_j e_{ij}. \tag{I.2}$$

This has rotational symmetry around the z-axis. The role of angular momentum transfer becomes obvious if we assume the F_{el} and F_{me} are isotropic. In this case, F_{el}, is expressed as

$$F_{\text{el}} = \frac{1}{2} C_{ijkl} e_{ij} e_{kl}, \tag{I.3}$$

where C_{ijkl} is the elastic constant and can be written as $C_{ijkl} = \lambda \delta_{ij} \delta_{kl} + \mu(\delta_{ik}\delta_{jl} + \delta_{il}\delta_{jk})$. Here λ and μ are Lame's elastic constants. F_{mag} is composed of the isotropic exchange interaction and magnetic anisotropy $-Km_z^2$. Because of the rotational symmetry around the z-axis, a phononic angular momentum J_{ph}^z is preserved in the elastic system and a magnetic angular momentum S_z is preserved in the magnetic system[1]. F_{me} connects these two systems preserving the total angular momentum $J_{\text{ph}}^z + S_z$. Thus, the F_{me} transfers angular momentum from phononic to magnetic system.

In reality, the rotational symmetry around the z-axis is broken by the interference between film and substrate and the magnetic field. The symmetry breaking may cause the decay of

---

* Present address:Center for Emergent Matter Science (CEMS), RIKEN, Wako 351-0198, Japan

[1] Here we ignored the orbital components of angular momenta [1, 2] because we regard the SAW as that circular polarized plane wave and consider the long wavelength limit.

angular momentum. This is similar to the case of spin momentum studied in the spintronics scientific field. Since the spin is not preserved quantity, the spin current is exponentially decayed with the spin diffusion length but still meaningful in the microscopic range. The phonon angular momentum may also show gradual decay. Nevertheless, the magnetization can be controlled by the angular momentum transfer if the magnitude is large enough to overcome the decay.

The angular momentum transfer via magnetoelastic coupling is more clearly shown by the quantum expression as follows. As shown in the main text, the magnetoelastic coupling $F_{\rm me}$ can be written as

$$F_{\rm me} = \frac{b(g\mu_{\rm B})^2}{2}\Big[S_x(S_+e_{x-} + S_-e_{x+}) + 2S_y^2 e_{yy}\Big]. \tag{I.4}$$

According to this equation, $F_{\rm me}$ does transfer the angular momentum if e_{x+} and e_{x-} raise and lower the quantum number of z-axis angular momentum. To demonstrate this, we consider the second quantization of the displacement vector

$$\begin{aligned}\mathbf{u}(\mathbf{r}) &= \sum_{\boldsymbol{k},n}\sqrt{\frac{\hbar}{2\omega_{\boldsymbol{k},n}N}}\Big(\epsilon^n_{\boldsymbol{k}} a^n_{\boldsymbol{k}} e^{i(\boldsymbol{k}\cdot\boldsymbol{r}-\omega_{\boldsymbol{k},n}t)} + h.c.\Big)\\ &= \sqrt{\frac{\hbar}{2\omega_0 N}}\Big(\epsilon_+ a_+ e^{i(k_0x-\omega_0t)} + h.c.\Big) + \sqrt{\frac{\hbar}{2\omega_0 N}}\Big(\epsilon_- a_- e^{i(-k_0x-\omega_0t)} + h.c.\Big)\\ &+ \sum_{\rm others}\sqrt{\frac{\hbar}{2\omega_{\mathbf{k}',n'}N}}\Big(\epsilon^{n'}_{\mathbf{k}'} a^{n'}_{\mathbf{k}'} e^{i(\mathbf{k}'\cdot\mathbf{r}-\omega_{\mathbf{k}',n'}t)} + h.c.\Big),\end{aligned} \tag{I.5}$$

where $\epsilon^n_{\mathbf{k}}$ is the polarization of n-th acoustic wave mode at wave vector $\mathbf{k}$ and $\epsilon_\pm = (1, \pm i, 0)/\sqrt{2}$. In the right-hand side, we discriminate two circular polarization modes, which we regard as counter-propagating SAW modes, and other acoustic waves. ω_0 and k_0 are the frequency and wave vector of the SAW, respectively. $a^n_{\mathbf{k}}$ and $a^{n\dagger}_{\mathbf{k}}$ are phonon annihilation and creation operators for n-th acoustic wave mode at wave vector $\mathbf{k}$. $a_\pm$ and $a^\dagger_\pm$ are annihilation and creation operators of SAW, respectively. In the following, we only describe the SAW acoustic waves and neglect the last term of the above equation.

The z-component of phonon angular momentum $\mathbf{J}_{\rm ph} = \sum_l \mathbf{u}_l \times \dot{\mathbf{u}}_l$ of SAW mode is given by[3]

$$J^z_{\rm ph} = \hbar\Big(n_+ + \frac{1}{2}\Big) - \hbar\Big(n_- + \frac{1}{2}\Big). \tag{I.6}$$

Here, $n_+ = a^\dagger_+ a_+$ and $n_- = a^\dagger_- a_-$ represent the number operator the SAW mode with right- and left-handed circular polarizations, respectively. Since $e_{x\pm} = e_{xx} \pm i(2e_{xy}) =$

$\partial_x u_x \pm i(\partial_x u_y + \partial_y u_x)$, the quantum operators $e_{x\pm}$ are written as

$$e_{x+} = -ik\sqrt{\frac{\hbar}{2\omega_0 N}}\left(a_+^\dagger e^{-i(k_0x-\omega_0t)} + a_- e^{-i(k_0x+\omega_0t)}\right), \tag{I.7}$$

$$e_{x-} = ik\sqrt{\frac{\hbar}{2\omega_0 N}}\left(a_+ e^{i(k_0x-\omega_0t)} + a_-^\dagger e^{i(k_0x+\omega_0t)}\right). \tag{I.8}$$

Thus, we get

$$e_{x+}\left|n_+\right\rangle = -ik_0\sqrt{\frac{\hbar}{2\omega_0 N}}e^{-i(k_0x-\omega_0t)}\sqrt{n_+ + 1}\left|n_+ + 1\right\rangle, \tag{I.9}$$

$$e_{x-}\left|n_+\right\rangle = ik_0\sqrt{\frac{\hbar}{2\omega_0 N}}e^{i(k_0x-\omega_0t)}\sqrt{n_+}\left|n_+ - 1\right\rangle, \tag{I.10}$$

$$e_{x+}\left|n_-\right\rangle = -ik_0\sqrt{\frac{\hbar}{2\omega_0 N}}e^{-i(k_0x+\omega_0t)}\sqrt{n_-}\left|n_- - 1\right\rangle, \tag{I.11}$$

$$e_{x-}\left|n_-\right\rangle = ik_0\sqrt{\frac{\hbar}{2\omega_0 N}}e^{i(k_0x+\omega_0t)}\sqrt{n_- + 1}\left|n_- + 1\right\rangle, \tag{I.12}$$

where $|n_\pm\rangle$ represent the phonon number states of these SAW, respectively. This clearly represents the $e_{x\pm}$ operators raise or lower the quantum number of circular polarized acoustic wave. In other words, these increase or decrease the phonon angular momentum by $\hbar$. This shows that the magnetoelastic coupling transfer the angular momentum from elastic to magnetic system. In the present case, the excited SAW is a macroscopic wave. Even in that case, the acoustic excitation of magnetic resonance can be described as many superpositions of angular momentum transfer quantum process.

II. ANALYSIS OF SAW TRANSMISSION SPECTRA

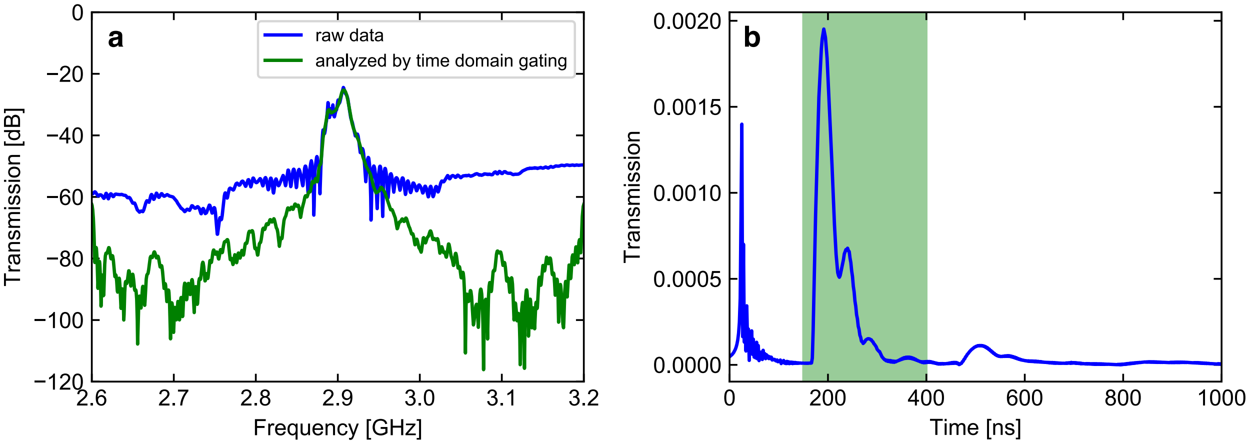

FIG. S1. **Raw and analyzed SAW transmission spectra.** **a**, A raw transmission spectrum and the analyzed spectrum by using time domain gating. **b**, The transmission of the SAW device in time domain obtained by inverse Fourier transform.

Figure S1a exemplified the absolute values of raw and analyzed transmission spectra from IDT 1 to IDT 2 (S_{21}) measured by a vector network analyzer. Not only the signal carried by SAW propagation but also the direct microwave crosstalk contribute to the raw spectra. To remove the direct crosstalk contribution, we performed the time domain gating as follows. By inverse Fourier transform of the transmission in frequency domain, we get the transmission as a function of time. Figure S1b shows the absolute value of the transmission in time domain. The narrow peak at time near 0 ns is attributed to the direct microwave crosstalk between two IDTs. The transmission peaks around 170 ns are the main signal owing to SAW propagation. The small peak at 500 ns is caused by the multiple reflections from IDTs. By Fourier transforming the time domain transmission only within the time region of the main SAW signal (150 - 400 ns), we obtained the SAW transmission spectrum (green line in Fig. S1a), in which the direct crosstalk and the contribution of multiple reflection are removed.

The transmissions $T_{+k}(H)$ presented in the main text is defined as $T_{+k}(H) = \bar{S}_{21}(H)/\bar{S}_{21}(400\ \mathrm{mT})$, where $\bar{S}_{21}$ is the average of time gated S_{21} spectrum between 2.8 GHz and 3.0 GHz in the unit of V. We similarly obtained the $T_{-k}(H) = \bar{S}_{12}(H)/\bar{S}_{12}(400\ \mathrm{mT})$, where $\bar{S}_{12}$ is the average of time gated S_{12} spectrum.

III. SAW TRANSMISSION AND NONRECIPROCITY IN THE MAGNETIC FIELDS ALONG VARIOUS IN-PLANE DIRECTIONS

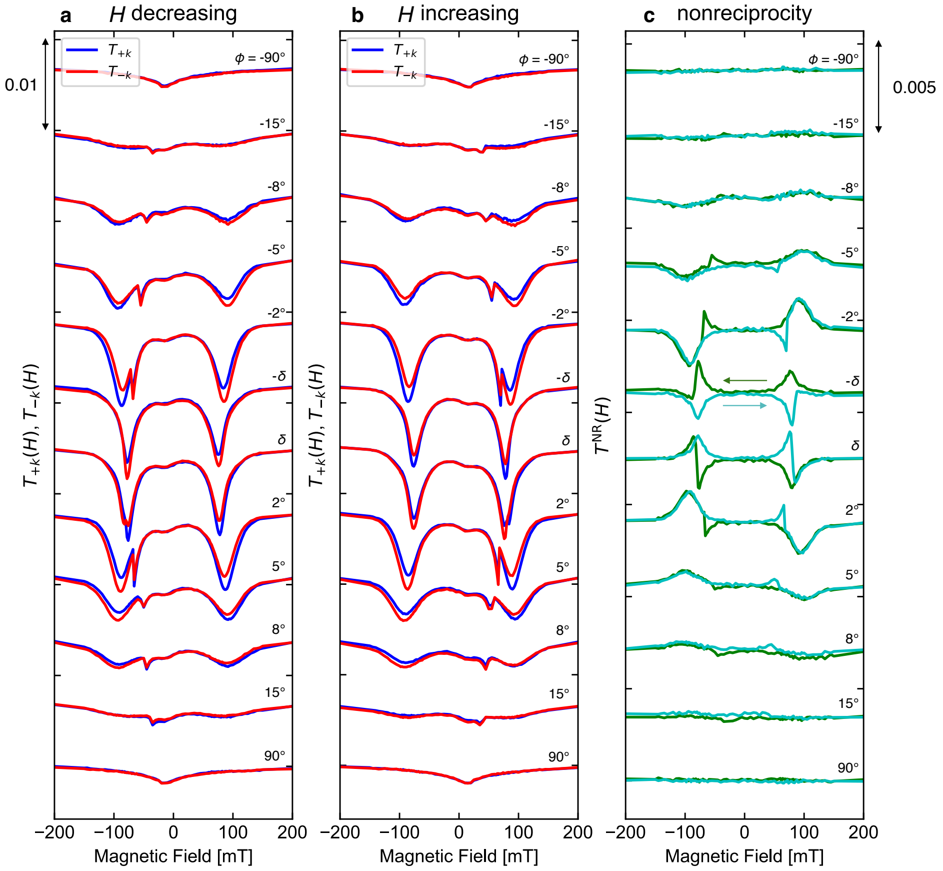

FIG. S2. **SAW transmission and nonreciprocity in magnetic fields parallel to various in-plane directions a,b**, SAW transmission along $+x$ and $-x$ directions $T_{+k}(H)$, $T_{-k}(H)$ in decreasing (**a**) and increasing (**b**) magnetic fields applied at various angles ϕ. **c**, nonreciprocity $T^{\mathrm{NR}}(H) = T_{+k}(H) - T_{-k}(H)$ in decreasing (green) and increasing (cyan) the magnetic field at various angle ϕ. The $+\delta$, $-\delta$ are positive and negative angles very close to 0 deg. The deviation is less than 0.5°. Initially, we adjust the rotator as close to zero as possible. Judging from the observed SAW transmission and magnetoresistance, the angle is slightly tilted to the positive side. $+\delta$ denotes this angle. After that, we rotated to the negative direction by 0.5°. Then, the angle seems to be slightly negative. We define this angel as $-\delta$. It is difficult to get ϕ closer to zero reproducibly. Hereafter, we use these notations for the angles very close to zero.

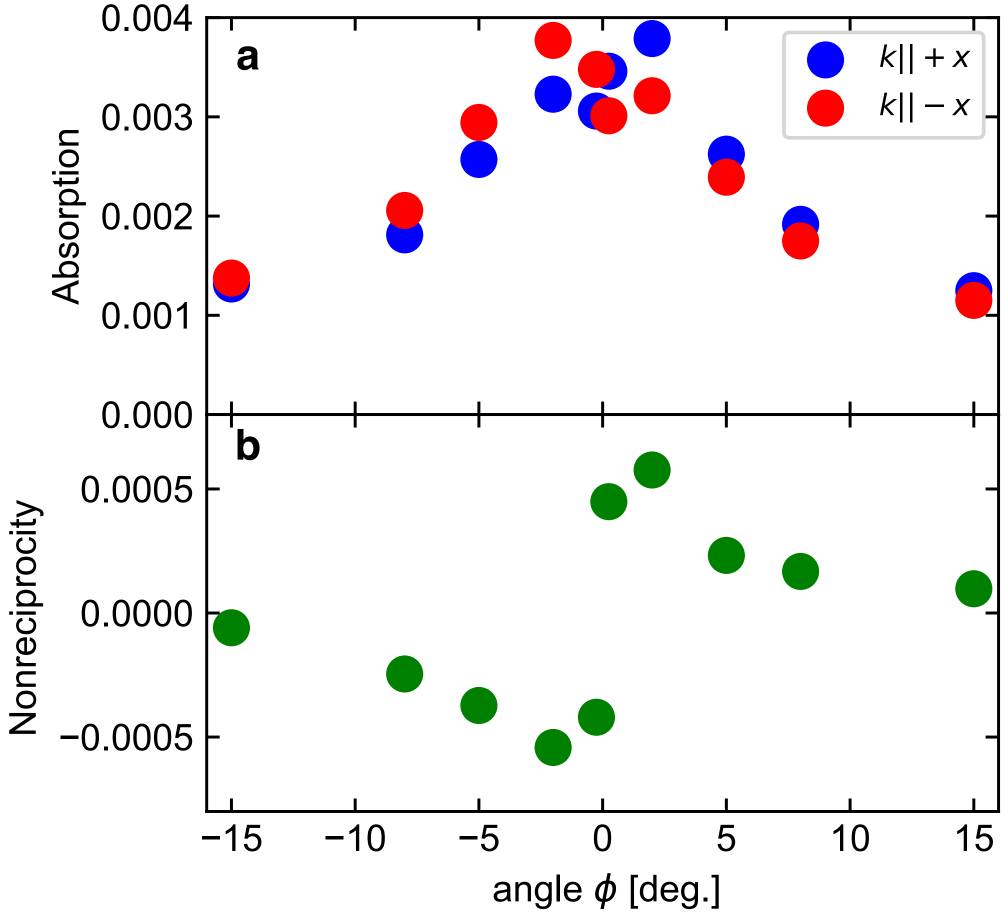

FIG. S3. **The angle ϕ dependence of the SAW absorption and nonreciprocity.** **a**, ϕ dependence of the absorption of the SAW with the wave vectors k parallel ($k||+x$) and antiparallel ($k||-x$) to the x-axis in decreasing the magnetic field. The SAW absorption is calculated by averaging $-T_{+k}(H)$ and $-T_{-k}(H)$ in the region of $H > 0$ for decreasing H at each ϕ. **b**, ϕ dependence of the nonreciprocity estimated by the difference of absorptions for $k||x$ and $k||-x$.

IV. PHASE DEPENDENCE

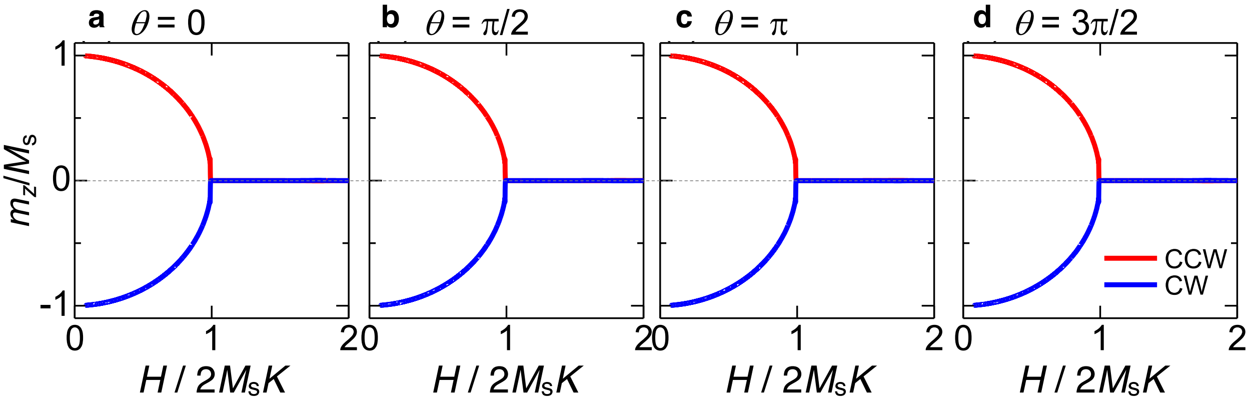

FIG. S4. **SAW phase dependence of magnetization evolution under SAW poling. a,b,c,d** The numerically calculated magnetic field dependence of magnetization under the application of SAW currents along $+x$ and $-x$ with various SAW initial phases $\theta = 0$ (**a**), $\pi/2$ (**b**), π (**c**), $3\pi/2$ (**d**).

The magnetization control in this work is totally different from the so-called precessional magnetization switching[4], in which the final magnetization direction depends on the phase of precession. To demonstrate the difference, we show in Fig. S4 the calculated magnetization evolution under the acoustic wave with clock wise (CW) and counter clockwise (CCW) polarization with various SAW initial phases θ at $t = 0$. It clearly revels that switching is independent of the phase but depends only on polarization, being consistent with the picture of angular momentum transfer.

V. ANISOTROPIC MAGNETORESISTANCE OF NI FILM.

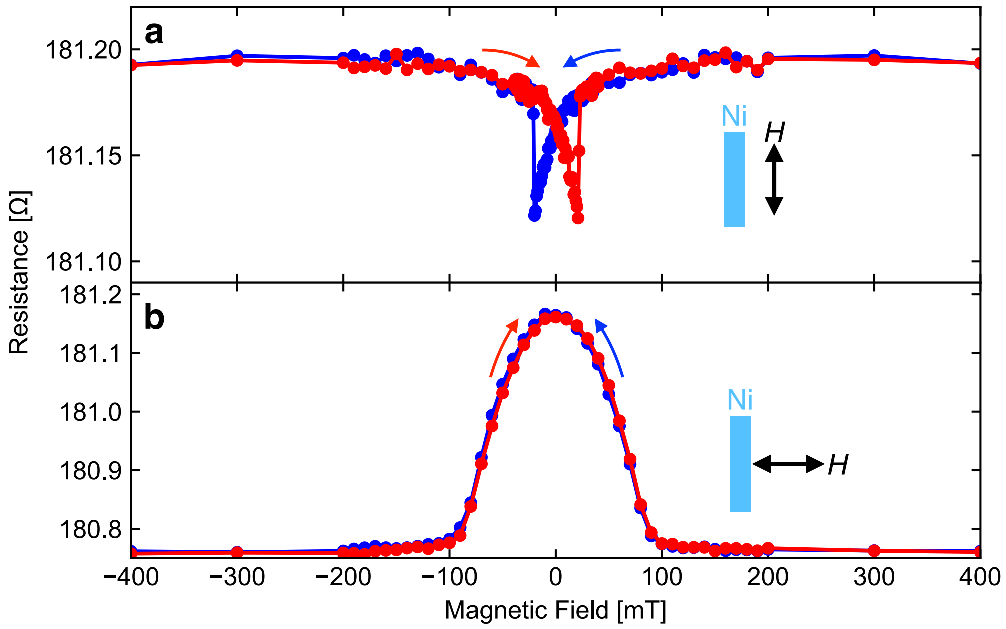

FIG. S5. **Anisotropic magnetoresistance of Ni film.** **a**, The resistances in magnetic fields parallel to the Ni film. The blue and red line shows those in decreasing and increasing magnetic fields. **b**, The resistances in magnetic fields perpendicular to the Ni film. The blue and red line shows those in decreasing and increasing magnetic fields.

VI. MAGNETIZATION AFTER THE POLING WITH THE VARIOUS MAGNETIC FIELD ANGLES AND SAW EXCITATION MAGNITUDES AS PROBED BY MAGNETORESISTANCE

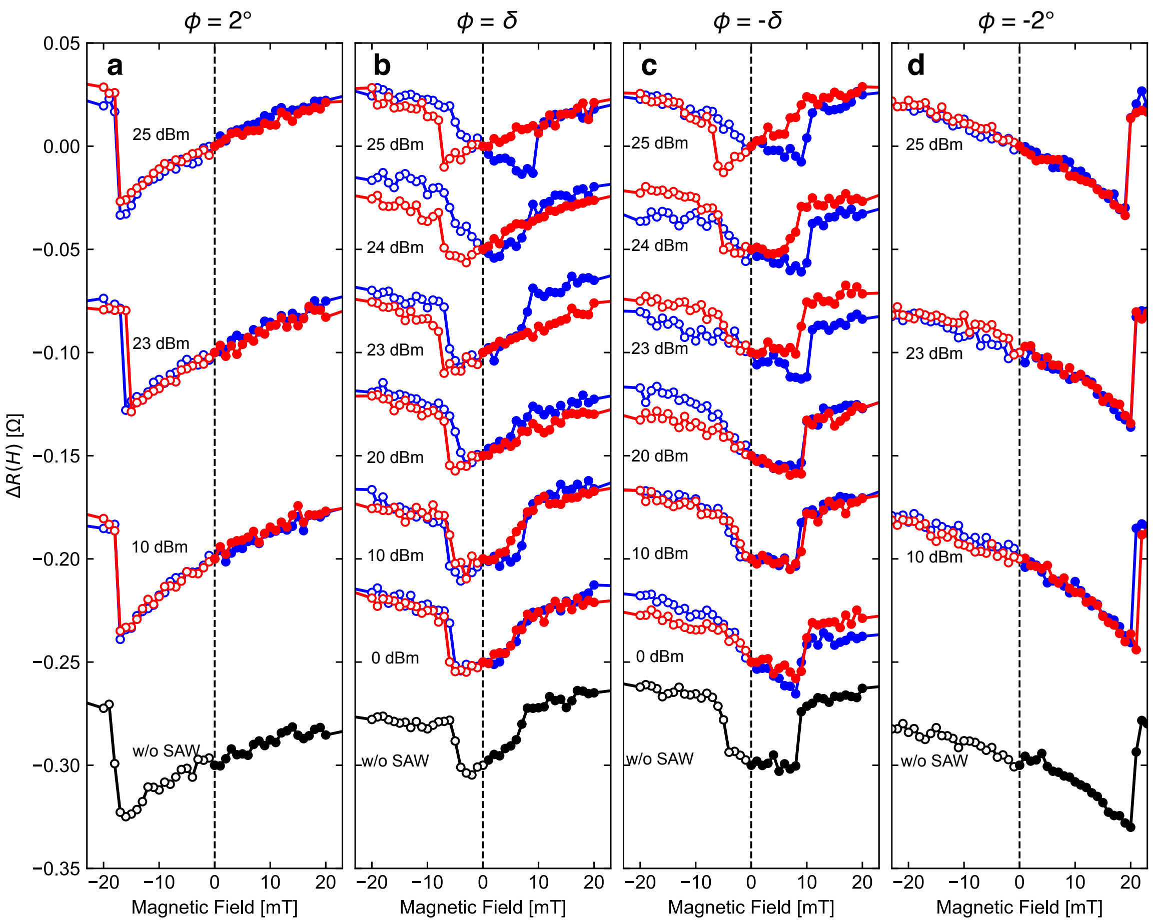

FIG. S6. **Magnetoresistance after poling with various magnetic field angles and SAW excitation intensities. a,b,c,d** Magnetoresistance $\Delta R(H) = R(H) - R(0)$ in the field parallel to the z-axis after the poling with various conditions. In the poling procedure, the magnetic field as large as 400 mT is initially applied, and SAW current is injected along x or $-x$ direction. Then the magnitude of magnetic field is decreased to 0 T. The angles between x-axis and poling magnetic field are $+2°$, $+\delta$, $-\delta$, $-2°$ in a, b, c, and d, respectively. The blue and red lines and circles represent the magnetoresistance after the poling with SAW current along $+x$ and $-x$, respectively. The black solid lines and circles show the magentoresistance after poling without SAW current. The black dashed line emphasizes the initial field of 0 T. The data presented in the main text is that of $\phi = \delta$ and 25 dBm.

Figure S6 shows the magnetoresistance $\Delta R(H) = R(H) - R(0)$ after the poling with various magnetic field angles ϕ and SAW excitation magnitudes. At $\phi = \pm\, 2°$, the magnetization direction after the poling is determined only by the tilting direction of the poling magnetic field, being insensitive to the direction and magnitude of SAW currents. The slope of magnetoresistance for $\phi = +2°$ ($\phi = -2°$) is positive (negative) and the discontinuous increase is observed at a negative (positive) magnetic field, indicating that the magnetization after the poling is along $+z$ ($-z$) direction. At $\phi = +\delta$ and $-\delta$ the magnetoresistance slightly increase and decrease around $H = 0$ T, respectively, and does not show any SAW current direction dependence in the cases of small SAW excitation. When the SAW excitation increases above 23 dBm, the magnetoresistance becomes dependent on SAW current direction. At 25 dBm, the slope of magnetoresistance seems governed by the sign of SAW direction.

VII. PROBING MAGNETIZATION DIRECTION BY NONRECIPROCITY

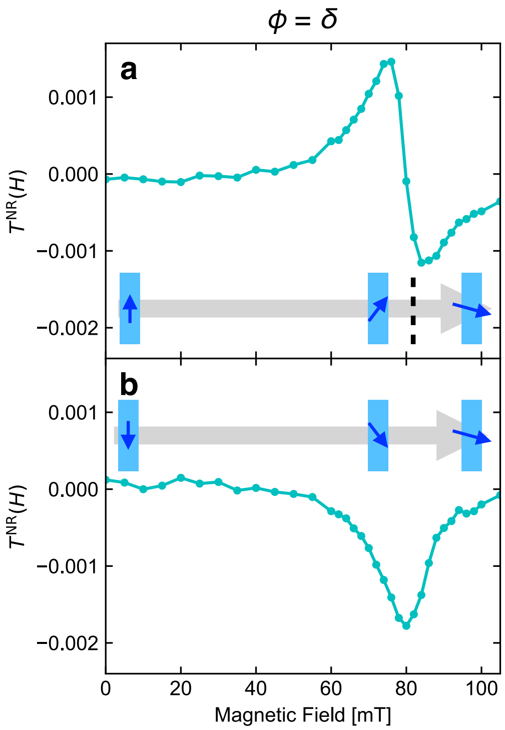

FIG. S7. **Nonreciprocity after the application of the magnetic field parallel to the Ni film. a,b**, The magnetic field dependence of the nonreciprocity at $\phi = \delta$ after the application of magnetic fields as large as 400 mT parallel to $-z$ (a) and $+z$ axes (b) without any SAW excitation. Insets illustrate the inferred magnetization directions. The black dashed line emphasize the magnetization θ flop.

After a strong magnetic field is applied parallel (antiparallel) to the z-axis and decreased to zero, the magnetization should point at $+z$ $(-z)$ direction. By using these states, we confirm that the measurement of SAW nonreciprocity certainly probe the magnetization direction after the poling. Figures S7a and b show variation of the nonreciprocity $T^{\mathrm{NR}}(H)$ in increasing the magnetic field at $\phi = \delta$ from 0 mT after the application and removal of magnetic fields as large as 400 mT antiparallel and parallel to z-axes, respectively. In measuring $T^{\mathrm{NR}}(H)$, the magnetic field is increased from 0 mT, and the microwave power is 10 dBm. $T^{\mathrm{NR}}(H)$ increases and decreases for the initial magnetization antiparallel and parallel to $+z$-axis, respectively. The sign change due to the magnetization θ flop was discerned only in Fig. S7a. These results confirm that the magnetic field variation of nonreciprocity

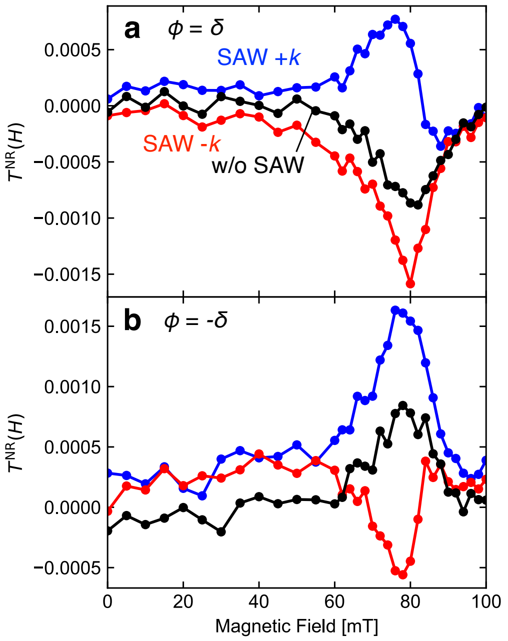

FIG. S8. **Nonreciprocity after the poling with SAW current. a,b**, The magnetic field dependence of the nonreciprocity $T^{\mathrm{NR}}(H)$ after the poling with the magnetic fields at $\phi = \delta$ (**a**) and $-\delta$ (**b**). The blue and red lines represent $T^{\mathrm{NR}}(H)$ after the poling with SAW currents parallel and antiparallel to the x-axis, respectively. The black lines show those after the poling without the SAW input.

certainly probe the direction of the magnetization at zero magnetic field.

Figures S8a and b show the magnetic field dependence of the nonreciprocity after the poling with or without SAW. In these cases, the poling and measurement magnetic fields have the same direction: $\phi = +\delta$ in Fig. S8a and $\phi = -\delta$ in Fig. S8b. The magnetization direction after the poling without the SAW (black line) seems determined by the tilted direction of the magnetic field in the poling process. On the other hand, in the presence of SAW current along $+x$ (blue line) and $-x$ (red line) directions, the magnetization direction seems governed by the SAW propagation direction in the poling procedure.

---

[1] Nakane, J. J. & Kohno, H. Angular momentum of phonons and its application to single-spin relaxation. *Phys. Rev. B* **97**, 174403 (2018).

[2] Yan, P., Kamra, A., Cao, Y. & Bauer, G. E. W. Angular and linear momentum of excited

ferromagnets. *Phys. Rev. B* **88**, 144413 (2013).

[3] Zhang, L. & Niu, Q. Angular Momentum of Phonons and the Einstein–de Haas Effect. *Phys. Rev. Lett.* **112**, 085503 (2014).

[4] Thevenard, L. *et al.* Precessional magnetization switching by a surface acoustic wave. *Phys. Rev. B* **93**, 134430 (2016).
