# Supplementary material for: Magnetization control by angular momentum transfer from surface acoustic wave to ferromagnetic spin moments
Source: Nat Commun. 2021 May 10;12:2599. doi: 10.1038/s41467-021-22728-6 (PMC8110840; doi:10.1038/s41467-021-22728-6)
Supplement: Supplementary file 2 — Peer Review File [file 41467_2021_22728_MOESM2_ESM.pdf]

Reviewers' Comments:

Reviewer #1:
Remarks to the Author:
In the manuscript NCOMMS-20-30034-T entitled "Magnetization control by angular momentum transfer from surface acoustic wave to ferromagnetic spin moments", the authors reported magnetization rotation assisted by angular momentum transfer from the surface acoustic wave (SAW) to magnetic system. They investigated the direction of magnetization rotation after applying the SAW by measuring the magnetoresistance and nonreciprocal SAW transmission. They observed that the direction of magnetization rotation could be controlled by changing the polarity of SAW current. Overall, the paper includes clear results and argumentation, I cannot recommend this paper for publication in Nature Communications due to the following reasons:

My main concerns are the novelty of the manuscript and quantum mechanical interpretation of the angular momentum of the SAW.

(i) The magnetoelastic effect whose Hamiltonian is given by Eqs. (2) and (3) has been reasonably established. Moreover, the angular momentum transfer from the SAW has been already demonstrated in the non-reciprocal spinwave excitation which has been reported by same authors (PRB 95, 020407 (2017)). The concern here is it is not clear how this work sets itself aside from previously reported work.

(ii) The authors claim that the SAW provides quantum phonon angular momentum which can be transferred to spin angular moment as described in Supplementary. However, the manuscript lacks the experimental evidence that the angular momentum of the SAW can be described by quantum mechanical operators. I believe that the SAW used in the experiment should be considered as classical acoustic wave rather than quantum mechanical one because the wavelength is longer than the mean free path of electrons in the metallic Ni and the fundamental frequency is lower than the electron relaxation frequency. Namely, the SAW does not interact with electron system via nucleation/annihilation process. Is the angular momentum transfer observed in the experiment simply owing to macroscopic local angular momentum conservation?

Reviewer #2:
Remarks to the Author:
Review
Magnetization control by angular momentum transfer from surface acoustic wave to ferromagnetic spin moments
by R. Sasaki, Y. Nii, and Y. Onose

In this manuscript, the authors demonstrate that the phonon angular momentum (PAM) of surface acoustic wave can control the magnetization of a ferromagnetic Ni film by means of the phononic-to-electronic conversion of angular momentum in a Ni/$LiNbO_3$ hybrid device. The result shows that the phonon angular momentum is useful for increasing the functionality of spintronic devices. The results are very interesting and significant. To be accepted by Nature Communication, the authors must address the following questions:

1. The authors states that the PAM of surface acoustic wave (SAW) can control the magnetization, how much about the PAM generated by the SAW? In the supplementary information, they only assume these SAW states has perfectly circular polarization, how about the general SAW they used in this experiment? Whether can they control the PAM of SAW?
2. The authors should adjust the PAM of SAW, then discuss how does the magnitude of PAM affect the magnetization.
3. Can they show more experimental evidence on the process of phononic-to-electronic conversion

of angular momentum, is there conservation law of PAM? Such discussion is better to be included in the revised version.

4. The authors claim that the PAM is transferred by the strain tensor, can we apply a kind of other static strain then also control the magnetization of a ferromagnetic Ni film?

Reviewer #1:

Author's response to the comments of Reviewer #1

Followings are our straightforward response to the comments.

(i)The magnetoelastic effect whose Hamiltonian is given by Eqs. (2) and (3) has been reasonably established. Moreover, the angular momentum transfer from the SAW has been already demonstrated in the non-reciprocal spin wave excitation which has been reported by same authors (PRB 95, 020407 (2017)). The concern here is it is not clear how this work sets itself aside from previously reported work.

Answer to (i)
As the reviewer suggested, Eqs. (2) and (3) are well-known. Nevertheless, we do not agree with the statement that the phenomena derived from well-known mechanisms are all trivial. If this were true, studies in applied science fields, which are based on well-known mechanisms, would be all unimportant. In fact, most of the results in condensed matter physics are also based on the well-known Schrödinger equation.
Regrading our previous paper, we would like to stress that the nonreciprocal propagation of the surface acoustic wave (SAW) is totally different from the main result of the present paper, which is magnetization control. While there is some similarity in the microscopic mechanism, we believe that the totally new macroscopic phenomenon of magnetic control should have a strong impact because magnetization control is the central issue of spintronics and spin-related physics.

(ii) The authors claim that the SAW provides quantum phonon angular momentum which can be transferred to spin angular moment as described in Supplementary. However, the manuscript lacks the experimental evidence that the angular momentum of the SAW can be described by quantum mechanical operators. I believe that the SAW used in the experiment should be considered as classical acoustic wave rather than quantum mechanical one because the wavelength is longer than the mean free path of electrons in the metallic Ni and the fundamental frequency is lower than the electron relaxation frequency. Namely, the SAW does not interact with electron system via nucleation/annihilation process. Is the angular momentum transfer observed in the experiment simply owing to macroscopic local angular momentum conservation?

Answer to (ii)
As the reviewer suggested, the magnetization control by the SAW angular momentum is a macroscopic phenomenon. The transferred energy and angular momentum are quite large compared with the quantized values. In this sense, this might be regarded as "classical" phenomena.
Nevertheless, all phenomena in this world can be described based on quantum mechanics. Macroscopic phenomena are composed of the superposition of many quantum processes while the quantum coherence is lost. Our result is not related to quantum coherence and therefore the quantum explanation is still effective. The main concept of this paper is angular momentum transfer. This is commonly valid in quantum and classical mechanics. We present the quantum mechanical explanation simply because the description is easier. To address this point, we revised the first section of supplementary information.
Incidentally, the mean free path and relaxation time are unrelated to the quantum condition in this case because our system are mainly composed of elastic wave and magnetic moments. The electronic transport is irrelevant.

All the changes made are listed below:

1. We have totally revised the 1st section in the Supplemental Information, to respond to the 2nd comment of Reviewer#1 and the 3rd comment of Reviewer#2.
2. To respond to the 1st comment of Reviewer #2, we have added the description at 9th line in the right column of page 2.
3. To respond to the 2nd comment of Reviewer #2, we have added the description at 31st line in the left column of page 4.

4. To respond to the 3rd and 4th comments of Reviewer #2, we have added the description at 24th line in the left column of page 4.
5. Following the style of Nature Communications, we omitted the citation in the abstract, added heading s and subheadings of the sections. We added Data and Code availability sections and changed Reference section position. We modified the citation style in References.
6. To avoid a misunderstandings of controlled magnetization direction, we changed both the sign definition of magnetic field and field rotation angle for plot of Fig. 3 (c), (e), (f) and Supplementary Fig. 5 and 6 so that the positive magnetic field points +z direction. Correspondingly, we have added a sentence at the 6th line in the left column of page 4 and changed the sign of field rotation angle to the positive one (phi=90 deg) in the caption of Fig. 3.
7. We rewrite the Eq. (3) using spin moment S emphasizing the angular momentum transfer. The text is revised accordingly.

Reviewer #2:

Author's response to the comments of Reviewer #2

First of all, we thank the referee for valuable comments. Followings are our straightforward response to them.

1.The authors states that the PAM of surface acoustic wave (SAW) can control the magnetization, how much about the PAM generated by the SAW? In the supplementary information, they only assume these SAW states has perfectly circular polarization, how about the general SAW they used in this experiment? Whether can they control the PAM of SAW?

Answer to 1
The magnitude of phonon angular momentum (ellipticity of SAW polarization) can be estimated by the SAW nonreciprocity. Only the right-handed circular polarized mode of SAW can excite the ferromagnetic resonance. The left-handed mode is insensitive to the magnetic resonance. Because the wave vector reversal changes the rotational direction of SAW polarization, the non-reciprocal ratio reflects the polarization ellipticity.
In the present case, the nonreciprocal ratio is about 10 %. Prof. Nozaki group reported that the nonreciprocity can be controlled by thickness of thin film. (ref. Phys. Rev. Applied **13**, 034074 (2020)).
To address this point, we added the description at the 9th line in the right column of the page2.

2. The authors should adjust the PAM of SAW, then discuss how does the magnitude of PAM affect the magnetization.

Answer to 2
In our experiment, the SAW controls whether m||+z or m||-z state is stabilized. Because these two states are completely degenerated, in principle, infinitesimal magnitude of SAW can control the magnetization. We have confirmed that orders of magnitude smaller SAW can still control the magnetization in our numerical calculation.
In reality, the deviation of the magnetic field angle from the x-axis lifts the degeneracy of these two states. For the magnetization control, the magnitude of angular momentum should be large enough to overcome the energy imbalance due to the angle deviation. To address this point, we have added the description at 31st line in the left column of page 4.
The detail angle and magnitude dependences are shown in the Supplemental Information section 4.

3. Can they show more experimental evidence on the process of phononic-to-electronic conversion of angular momentum, is there conservation law of PAM? Such discussion is better to be included in the revised version.

4. The authors claim that the PAM is transferred by the strain tensor, can we apply a kind of other static strain then also control the magnetization of a ferromagnetic Ni film?

Answer to 3 and 4

The control of magnetization depending on the propagation direction of surface acoustic waves cannot be explained by the effects of static strains or other trivial effects, but it is naturally explained by the effects of phonon angular momentum transfer. To emphasize this point, we added the description at the 24th line in the left column of page 4.

As already mentioned in the introduction, the spin current and spin-transfer torque are central concepts in the spintronics field while the spin angular momentum is not a conserved quantity. The spin current is exponentially decayed with the spin diffusion length but still meaningful in the microscopic range. The present results show that the phonon angular momentum can play a similar role.

If the system is uniform and has rotational symmetry, the phonon angular momentum is preserved. However, the effects of crystals and surfaces, and the magnetic field break the rotational symmetry, and strictly speaking, the conservation law of angular momentum is broken. Therefore, the phonon angular momentum should be decayed gradually but the angular momentum can still be transferred via the magnetoelastic coupling. In the present case, the magnitude of phonon angular momentum transfer seems to be large enough to overcome the gradual decay. The theoretical description in the Supplemental Information was totally revised to reflect these points.

All the changes made are listed below:

1. We have totally revised the 1st section in the Supplemental Information, to respond to the 2nd comment of Reviewer#1 and the 3rd comment of Reviewer#2.
2. To respond to the 1st comment of Reviewer #2, we have added the description at 9th line in the right column of page 2.
3. To respond to the 2nd comment of Reviewer #2, we have added the description at 31st line in the left column of page 4.
4. To respond to the 3rd and 4th comments of Reviewer #2, we have added the description at 24th line in the left column of page 4.
5. Following the style of Nature Communications, we omitted the citation in the abstract, added heading s and subheadings of the sections. We added Data and Code availability sections and changed Reference section position. We modified the citation style in References.
6. To avoid a misunderstandings of controlled magnetization direction, we changed both the sign definition of magnetic field and field rotation angle for plot of Fig. 3 (c), (e), (f) and Supplementary Fig. 5 and 6 so that the positive magnetic field points +z direction. Correspondingly, we have added a sentence at the 6th line in the left column of page 4 and changed the sign of field rotation angle to the positive one (phi=90 deg) in the caption of Fig. 3.
7. We rewrite the Eq. (3) using spin moment S emphasizing the angular momentum transfer. The text is revised accordingly.

Reviewers' Comments:

Reviewer #1:
None

Reviewer #2:
Remarks to the Author:
The authors have addressed all my comments, the manuscript can be now be accepted.

Reviewer #3:
Remarks to the Author:
The manuscript by Sasaki et al. describes the control of magnetization orientation in a thin magnetic film via the angular momentum transfer from a SAW. The authors use a combination of magnetic field sweeps and SAW propagation, referred to as poling procedure in the manuscript, to set the magnetization direction in one or the other orientation. The physics concept behind this is that the SAW has circularly polarized component and thus carries angular momentum, and that the sign of the angular momentum depends on the propagation direction of the SAW. This should enable to torque the magnetization and thus eventually set its orientation, using an appropriate combination of magnetic field orientation and SAW propagation direction in the poling procedure. The authors provide clear experimental evidence for this magnetization control process, together with a theoretical analysis/model. To the best of my knowledge, such a control of static magnetization orientation via SAW application has not been performed before. Moreover, the notion that angular momentum transfer from an acoustic wave to the magnetization can be used to control the magnetization is an important finding. I thus consider the results reported new and interesting.

However, it should also be stressed that a field sweep (the poling procedure) still is necessary. If magnetic field sweeps are needed, one could also simply adjust the magnetization orientation using the magnetic field. The authors should work out clearly what the advantage of their approach as compared to "simple" field sweeps would be. It furthermore would also be interesting to see if the application of SAW radiation alone allows to control the static M orientation. Can the authors comment on this in the manuscript?

The previous round of refereeing has addressed other important issues, which the authors have worked to resolve. In its present form, the manuscript is well written, and the figures are clear. In particular, the main results (compiled in Fig. 3) are clearly described. I have one further comment/suggestion which the authors should consider: On page 3, the authors state that the resistance can be used to distinguish the magnetization direction at zero magnetic field. This is not as simple, since anisotropic magnetoresistance depends on the square of the magnetization orientation, such that the resistance in positive magnetization saturation and in negative saturation is identical – as in fact also experimentally evident from Fig. 3(c). This also is true for R(H=0) – one cannot read out the magnetization orientation from R(H=0). Along the same lines, the magnetization hysteresis M(H) shown in Fig. 3(d) does not match with R(H) in panel (c). If M would only switch from +saturation to –saturation, the resistance would NOT change at all. So the magnetization orientation angle is crucial for the interpretation of the data. The authors in fact use this in their analysis of R(H) with increasing or decreasing field, which can indeed be taken as evidence for the evolution of the magnetization orientation, thus allowing to infer the M(H=0) orientation from a magnetic field sweep (the evolution of R with H). I would urge the authors to take this up in their manuscript and also correct Fig. 3(d).

Taken together -- with appropriate changes to resolve the points raised above – I feel that the manuscript should be considered for publication in Nature Communications.

Ni film?

Reviewer #3:

Author's response to the comments of Reviewer #3

First of all, we thank the referee for valuable comments. Followings are our straightforward response to them.

1.However, it should also be stressed that a field sweep (the poling procedure) still is necessary. If magnetic field sweeps are needed, one could also simply adjust the magnetization orientation using the magnetic field.
(1-A) The authors should work out clearly what the advantage of their approach as compared to "simple" field sweeps would be.
(1-B) It furthermore would also be interesting to see if the application of SAW radiation alone allows to control the static M orientation. Can the authors comment on this in the manuscript?

Answer to 1-A
The advantage of our approach is that the information of the phonon propagation direction or microwave input port can be converted to that of magnetization direction. Such conversion cannot be achieved by simple field sweeps. We believe that this type of device may provide a direct interface between microwave telecommunication and magnetic storage.

Answer to 1-B
We believe that SAW application alone can control the static magnetization if the effective magnetic field of SAW is large enough. Enhancement of effective magnetic field can be achieved by the optimization of material and device structure. In fact, similar optimization makes the closely related phenomenon of nonreciprocal SAW propagation gigantic. After we first reported the nonreciprocal propagation of SAW in 2017 (Sasaki, R. et al., PRB (2017)), several studies have reported much larger nonreciprocity in improved devices (Xu, M. et al., Sci. Adv. (2020), Tateno, S. and Nozaki, Y PRApplied, (2020)). Similar optimization in material and devise structure may realize the magnetization control only by SAW.

To address these points, we added the description at the 40th line in the left column of the page 5.

2. The previous round of refereeing has addressed other important issues, which the authors have worked to resolve. In its present form, the manuscript is well written, and the figures are clear. In particular, the main results (compiled in Fig. 3) are clearly described. I have one further comment/suggestion which the authors should consider:
On page 3, the authors state that the resistance can be used to distinguish the magnetization direction at zero magnetic field. This is not as simple, since anisotropic magnetoresistance depends on the square of the magnetization orientation, such that the resistance in positive magnetization saturation and in negative saturation is identical – as in fact also experimentally evident from Fig. 3(c). This also is true for R(H=0) – one cannot read out the magnetization orientation from R(H=0). Along the same lines, the magnetization hysteresis M(H) shown in Fig. 3(d) does not match with R(H) in panel (c). If M would only switch from +saturation to –saturation, the resistance would NOT change at all. So the magnetization orientation angle is crucial for the interpretation of the data. The authors in fact use this in their analysis of R(H) with increasing or decreasing field, which can indeed be taken as evidence for the evolution of the magnetization orientation, thus allowing to infer the M(H=0) orientation from a magnetic field sweep (the evolution of R with H). I would urge the authors to take this up in their manuscript and also correct Fig. 3(d).

Answer to 2
As the reviewer suggested, M(H) in Fig. 3(d) does not match with R(H) in Fig. 3(c) in the previous version. We oversimplified the magnetization curve. According to R(H) data, the magnetization is saturated only in the large magnetic field region. As the magnetic field is decreased, the magnetization should gradually decrease before it shows a flop. In the previous version, we neglected the gradual decrease of M. We corrected Fig. 3(d), following the referee's comment. Text is modified accordingly.

All the changes made are listed below:

1. To respond to the 1st comment of Reviewer #3, we have added the description at the 40th line in the left column of the page 5.
2. To respond to the 2nd comment of Reviewer #3, we modified Fig. 3d and added the corresponding description in the main text at the 54th line in the right column of the page 3.

Reviewers' Comments:

Reviewer #3:
Remarks to the Author:
The authors have taken up all questions from the review process in the manuscript. With these changes, the manuscript now is eligible for publication.
